# Case Report: FAP^+^ fibroblasts and SPP1^+^ macrophages in SMARCA2-deficient while SMARCA4-preserved poorly differentiated lung adenocarcinoma: two case reports and multi-omics analysis

**DOI:** 10.3389/fimmu.2025.1568556

**Published:** 2025-05-16

**Authors:** Zhaoxuan Wang, Junying Wang, Shengmin Wang, Weijiao Xu, Yixiang Zhang

**Affiliations:** ^1^ Department of Thoracic Surgery, The First Affiliated Hospital of Dalian Medical University, Dalian, China; ^2^ Department of Pathology, The First Affiliated Hospital of Dalian Medical University, Dalian, China; ^3^ Department of Thoracic Surgery, Shanghai Chest Hospital, Shanghai Jiao Tong University School of Medicine, Shanghai, China

**Keywords:** mSWI/SNF, SMARCA2, SMARCA4, lung adenocarcinoma, tumor microenvironment

## Abstract

Two ATPase subunits, SMARCA4 (which encodes BRG1) and SMARCA2 (which encodes BRM), facilitate this process by hydrolyzing ATP to energize the activity of the mammalian switch/sucrose-non-fermenting (mSWI/SNF) complexes. Clinically, SMARCA4-deficient non-small cell lung carcinoma (SMARCA4-dNSCLC) were associated with the poorly differentiated histologic manifestations and poor prognosis. However, NSCLC exhibited the similar poorly differentiated features but loss of SMARCA2 and retained SMARCA4 have so far been underrecognized. Here, we reported two cases of poorly differentiated tumors with loss of SMARCA2 expression while preserved SMARCA4 expression and provided the morphologic, immunohistochemical, and genetic characterization of these tumors, which both arose in elderly male and appeared as the pulmonary lesion. Furthermore, we perform a comprehensive multi-omics analysis of the transcriptomic cohort GSE31210 (n=226), the proteomic cohort from the study by Chen et al. (n=89), and multiplexed immunohistochemistry (IHC) staining of these two cases to decipher the poor prognosis dependent on the immunosuppressive barrier formed by FAP^+^ fibroblasts and SPP1^+^ macrophages in the SMARCA2-deficient while SMARCA4-preserved poorly differentiated lung adenocarcinoma (LUAD). The report provides novel insights into the distinct roles of SMARCA2 and SMARCA4 in LUAD pathogenesis, highlighting the immunosuppressive tumor microenvironment associated with SMARCA2 deficiency.

## Introduction

The mammalian switch/sucrose-non-fermenting (mSWI/SNF) complexes regulate chromatin structure by adjusting DNA-nucleosome interactions, thereby increasing chromatin accessibility (1). The mSWI/SNF complexes are composed of 11 to 15 subunits assembled from the products of 29 distinct genes, resulting in three major forms: cBAF, PBAF, and ncBAF. Acting as tumor suppressors, these complexes have been found to harbor mutations in at least one of their subunits in 20% of human cancers. Loss or inactivation of mSWI/SNF components disrupts chromatin remodeling, impairs transcriptional regulation, and contributes to tumorigenesis by enabling epigenetic plasticity, dedifferentiation, and resistance to cell death. Among the subunits, SMARCA4 (BRG1) and SMARCA2 (BRM) function as the mutually exclusive ATPases that drive nucleosome repositioning, and are frequently altered in thoracic and genitourinary malignancies (2). Previous studies have reported that SMARCA4 mutations occur with varying frequencies across different cancer types, including approximately 15% in Burkitt’s lymphoma, 10–35% in non-small cell lung carcinoma (NSCLC), and 5–10% in medulloblastoma and melanoma (3–6). By contrast, SMARCA2 mutations are rare, though sporadically observed in lymphoma, leukemia, clear cell renal cell carcinoma, and gastric cancer (7). Notably, SMARCA2 downregulation has been detected in approximately 15% of solid tumors, primarily via reversible epigenetic mechanisms (8). In addition, the reduced expression or loss of other mSWI/SNF subunits, such as ARID1A (9), ARID2 (10), and SMARCB1 (11), correlates with poor differentiation, higher TNM stages, and unfavorable prognoses, suggesting the presence of additional subtypes within the mSWI/SNF-deficient thoracic tumor category.

In the context of thoracic and lung tumors, recent guideline and studies indicated that SMARCA4-deficient tumors, including SMARCA4-deficient undifferentiated tumors (SMARCA4-dUTs) and SMARCA4-deficient non-small cell lung carcinoma (SMARCA4-dNSCLC) were primarily recognized subtypes of mSWI/SNF-deficient tumors. Further, SMARCA4-dUTs has been recognized as a separate entity that categorized as “Other epithelial tumors” in the 2021 World Health Organization (WHO) classification of lung tumors (12). SMARCA4-dUTs typically exhibited co-deficiency of SMARCA4 and SMARCA2, reflecting profound dedifferentiation and aggressive biological behavior. A study proposed that SMARCA2 participated in the dedifferentiation process from conventional NSCLC to SMARCA4-dUTs, thereby contributing to the acquisition of aggressive phenotypes and resistance to therapy (13). Besides, studies by Metovic et al. (14) and Iwakoshi et al. (15) demonstrated that SMARCA2 deficiency, coupled with SMARCA4 retention, displayed clinicopathological characteristics similar to those of SMARCA4-UTs. Given the known tumor-suppressive function of SMARCA2, its selective loss could have critical implications for tumor differentiation, angiogenesis, progression, and metastasis (16, 17). Specially, recent evidence suggests that SMARCA2 deficiency may independently drive oncogenic transcriptional programs through aberrant activation of cell cycle regulators and chromatin destabilization pathways (18). Collectively, these results highlighted the potential of SMARCA2 as a key mediator of dedifferentiation in lung cancer and raise the possibility that SMARCA2-deficient but SMARCA4-retained tumors may constitute a distinct molecular and clinical entity.

In the study, we reported two cases of poorly differentiated lung adenocarcinoma (LUAD) characterized by SMARCA2 deficiency with preserved SMARCA4 expression. Through integrated transcriptomic, proteomic, and spatial analyses, we delineated the distinct tumor ecosystem and uncover mechanisms underlying their aggressive behavior. Our findings highlighted poorly differentiated tumors with loss of SMARCA2 expression while preserved SMARCA4 expression maybe a potential subtype in the mSWI/SNF-deficient undifferentiated tumors of the thoracic region. Also, these indicated that the potential of SMARCA4 as a predictive biomarker in SMARCA2-deficient LUAD, especially considering its preserved expression and possible compensatory role in chromatin remodeling and immune evasion.

## Case presentation

**Figure 1:**
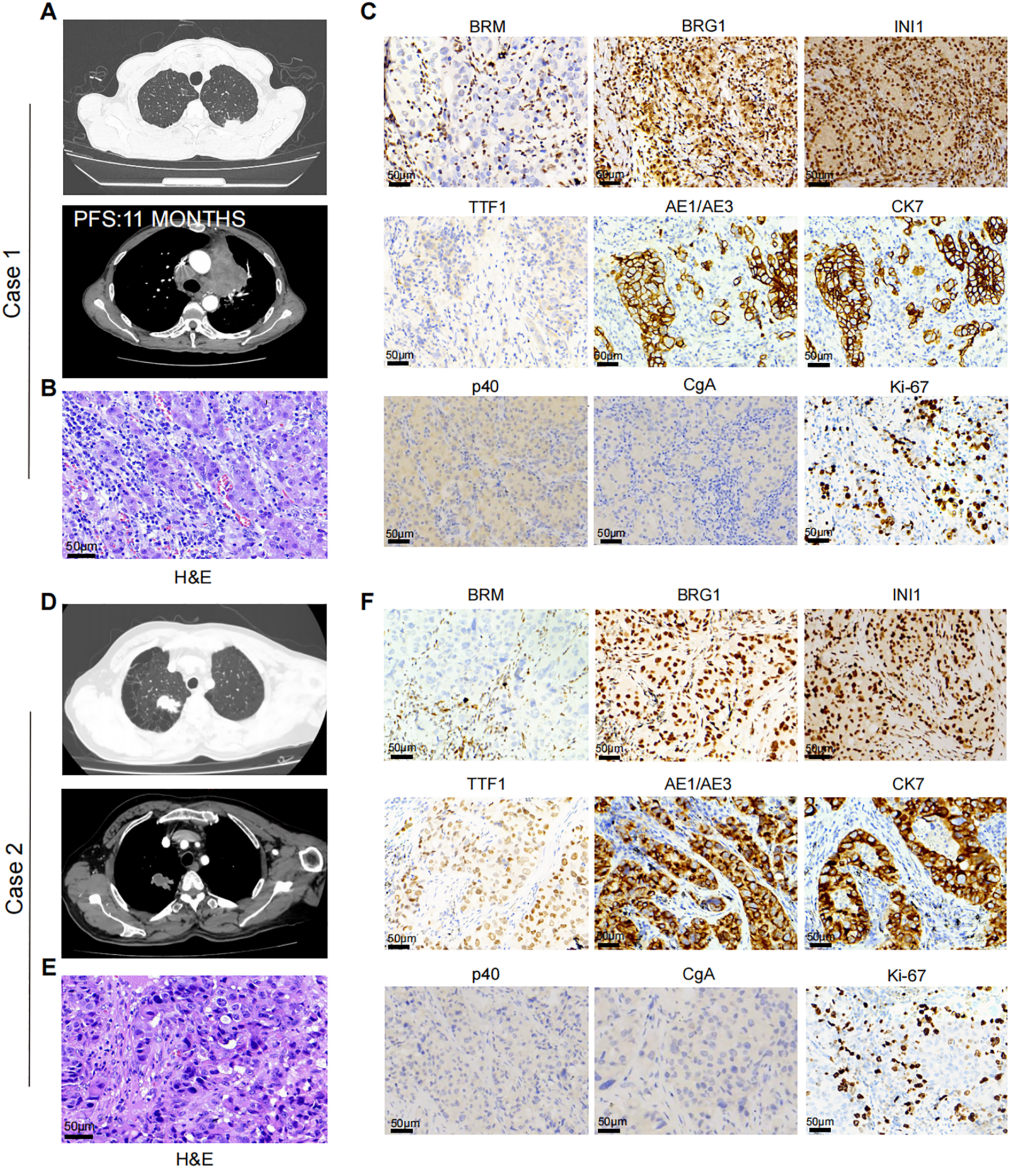
**(A)** Case 1. Chest CT-scan showed left upper lung lesion and recurrent para-aortic arch mass after 11 months. **(B)** Representative H&E staining of case 1. **(C)** Representative IHC staining showing the expression level of BRM, BRG1, INI1, TTF1, AE1/AE3, CK7, p40, CgA, and Ki-67 in Case 1. **(D)** Case 2. Chest CT-scan showed left upper lung lesion. **(E)** Representative H&E staining of case 1. **(F)** Representative IHC staining showing the expression level of BRM, BRG1, INI1, TTF1, AE1/AE3, CK7, p40, CgA, and Ki-67 in Case 2.

**Case 1:** A 66-year-old male presented to our thoracic department in July 2023 with multiple pulmonary nodules on a computed tomography (CT) scan during a routine check-up. He had a history of secondary pulmonary tuberculosis and a 40 pack-year smoking history. The chest CT scan revealed a solid subpleural lesion with a size of 2.6 cm × 1.3 cm in the left upper lobe, whereas all others were mixed or pure ground-glass nodules in the right upper lobe (**Figure 1A**). Perioperative laboratory tests revealed that serum CEA levels was 5.81 μg/L (reference range 0-5 μg/L), and other tumor markers including NSE, Cyfra21-1, and SCC were within the normal range. Head and abdomen CT showed no obvious abnormalities. Operative findings showed severe intrathoracic adhesions and a 3.2 cm × 2.8 cm tumor in the left upper lobe. He underwent the video-assisted thoracoscopic left upper pulmonary wedge resection and lymph nodes (Group 5 and 6) dissection. Unfortunately, the intraoperative frozen pathological examination revealed poorly differentiated carcinoma but legally authorized representative of the patient denied further open lobectomy. Postoperative H&E staining revealed that poorly differentiated adenocarcinoma invaded the visceral pleura but no lymph nodes metastasis (**Figure 1B**). Immunohistochemistry (IHC) staining revealed that BRM (-), BRG1 (+), INI1(+), p40 (-),TTF-1 (-), AE1/AE3 (+), CK7 (+), CgA (-), Ki-67 (70%) (**Figure 1C**). Next-generation sequencing (NGS) indicated no driver gene mutations. One month after surgery, the patient received 4 cycles (21 days/cycle) of carboplatin and pemetrexed adjuvant chemotherapy. No signs of recurrence were found during chemotherapy. In June 2024, the patient was admitted to the hospital due to epigastric pain. The contrast-enhanced chest CT showed a 4.0 cm × 3.9 cm mass in the para-aortic arch, and multiple enlarged lymph nodes in the mediastinum and bilateral supraclavicular regions (**Figure 1A**). Percutaneous biopsy of the mass suggested a poorly differentiated carcinoma. Laboratory tests revealed CEA was 15.00μg/L, and NSE, CYFRA21-1, and SCC were normal. Repeat NGS at the recurrence lesion indicated multiple somatic mutation, including ARIDIA (NM_006015.6): c.267_295del, STK11 (NM_000455.5): c.766G>T, CDKN2A (NM_000077.5): c.235dup, and NF1 (NM_00104249): c.6710del. Tumor mutation burden was 6.0Muts/Mb and PD-L1 tumor proportion score (TPS) > 50%. Progression-free survival (PFS) after the first-line treatment was 11 months. Then, the patient received second-line therapy with anti-PD-1 therapy (Camrelizumab). Regrettably, the patient could not tolerate second-line therapy, and finally denied further treatment.

**Case 2:** A 65-year-old male was admitted to our thoracic department in August 2024 due to accidental trauma. He had a smoking history of 50 pack-years. Chest CT scan revealed a 3.0 cm × 3.0 cm mass in the right upper lobe of the lung, with metastasis to the hilar lymph nodes (**Figure 1D**). Perioperative laboratory tests revealed that serum tumor markers were within normal range. Head and abdomen CT showed no obvious abnormalities. He underwent the video-assisted thoracoscopic right upper lobectomy and systematic mediastinal lymphadenectomy. Postoperative H&E staining revealed that poorly differentiated adenocarcinoma (**Figure 1E**). The IHC results are as follows: BRM (-), BRG1 (+), INI1(+), p40 (-), TTF-1 (-), AE1/AE3 (+), CK7 (+), CgA (-), Ki-67 (40%) (**Figure 1F**). Next-generation sequencing indicated no driver gene mutations and PD-L1 TPS ≥ 1%. One month after surgery, the patient received 4 cycles (21 days/cycle) of carboplatin and pemetrexed adjuvant chemotherapy. Until April 2025, no signs of recurrence were observed.

### Multi-omics analysis reveals FAP^+^ fibroblasts and SPP1^+^ macrophages induce poor prognosis in SMARCA2-deficient while SMARCA4-preserved poorly differentiated LUAD

**Figure 2:**
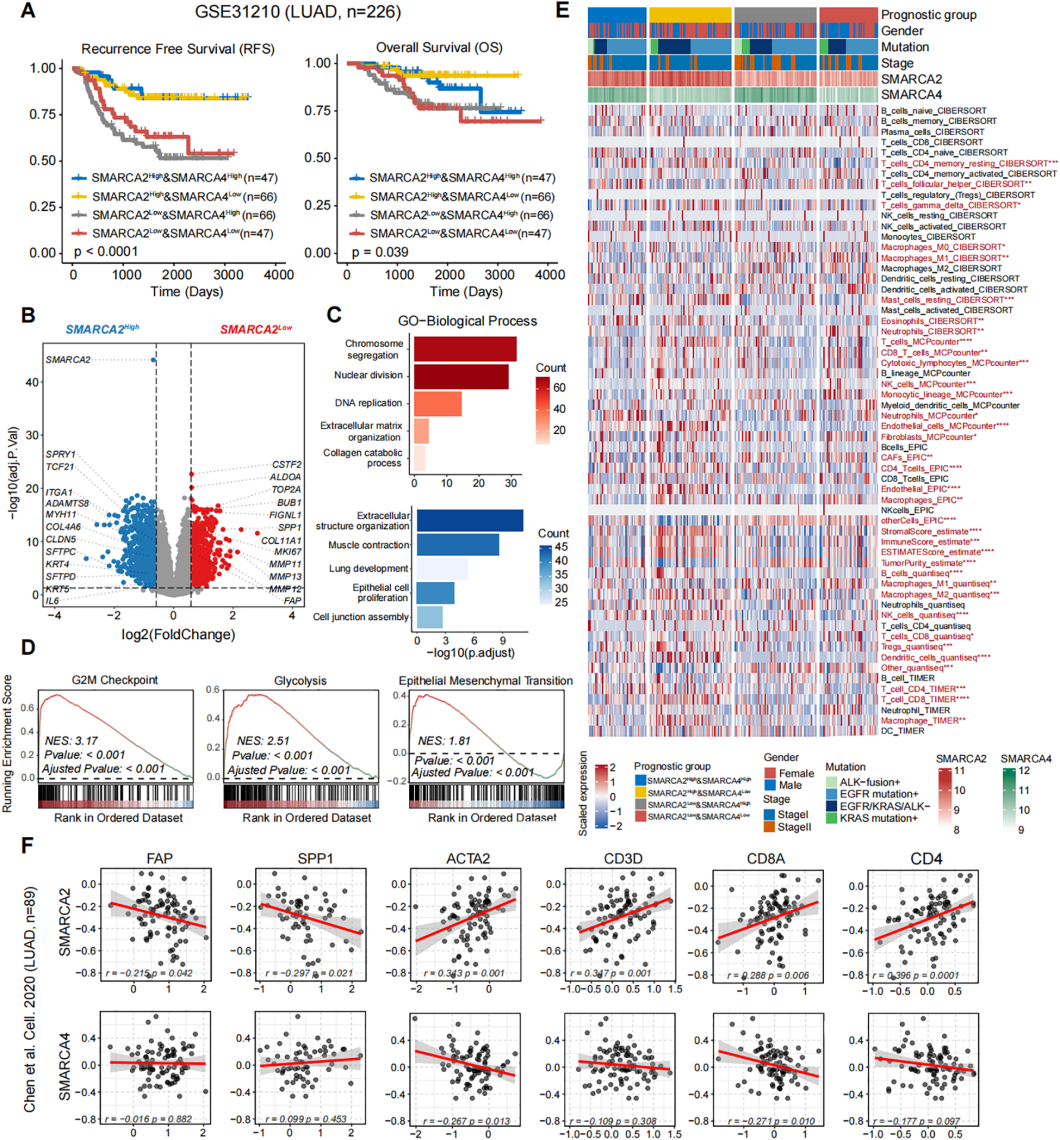
**(A)** Kaplan-Meier survival curves generated for four subgroups of LUAD patients stratified by the expression of SMARCA2 and SMARCA4 (median cutoff), and P-values were calculated by log-rank test. **(B)** The volcano plot of differential gene expressions in SMARCA2^Low^ versus SMARCA2^High^. The two vertical dashed lines represent absolute foldchange>1.5 in gene expression, and the horizontal dashed line denotes adjusted P-value cutoff 0.05. **(C)** GO-biological process (BP) and **(D)** GSEA enrichment analyses of DEGs in SMARCA2^Low^ and SMARCA2^High^. **(E)** Heatmap showing the infiltration of cell subpopulations for four subgroups of LUAD patients stratified by the expression of SMARCA2 and SMARCA4 (median cutoff) using multiple algorithms including CIBERSORT, MCP-counter, EPIC, ESTIMATE, quantiseq, and TIMER. Statistical significance was determined by Kruskal-Wallis’s test. **(F)** The scatterplot showing the correlation between the protein levels of SMARCA2 and SMARCA4 and FAP, SPP1, ACTA2, CD3D, CD8A, and CD4. Correlation analysis was created with Pearson’s correlation.

To further decipher the mechanisms of poor prognosis in SMARCA2-deficient while SMARCA4-preserved poorly differentiated LUAD, we analyzed the association between the expression levels of *SMARCA2* and *SMARCA4* and the prognosis in the transcriptomic LUAD cohort GSE31210 (n=226) (19). As expected, the results revealed that patients harboring *SMARCA2* ^Low^ (including *SMARCA2* ^Low^& *SMARCA4* ^High^ and *SMARCA2* ^Low^& *SMARCA4* ^Low^) exhibited shorter relapse-free survival (RFS) and overall survival (OS) (**Figure 2A**). Next, we analyzed the differentially expressed genes (DEGs) between the patients harboring *SMARCA2* ^Low^ and patients harboring *SMARCA2* ^High^ (including *SMARCA2* ^High^ & *SMARCA4* ^High^ and *SMARCA2* ^High^ & *SMARCA4* ^Low^). Genes correlated with cell cycle (*TOP2A*, *BUB1*, and *MKI67*) and glycolysis (*SLC2A1* and *ALDOA*) were significantly upregulated in patients harboring SMARCA2^Low^, whereas genes related to extracellular structure organization (*ADAMTS8*, *MYH11*, *COL4A6*, and *IL6*) and lung development (*SFTPD*, *TCF21*, and *SPRY1*) were upregulated in patients harboring *SMARCA2* ^High^ (**Figure 2B**). Gene ontology (GO) and gene set enrichment analysis (GSEA) analysis confirmed that nuclear division, G2/M checkpoint, and epithelial-mesenchymal transition (EMT) were markedly enriched in patients harboring *SMARCA2* ^Low^, which may elucidate the mechanism of higher proliferative ability and tendency of tumor recurrence (**Figures 2C, D**). Notably, we observed the overexpression of extracellular matrix (ECM) remodeling-related genes (*COL11A1*, *MMP12*, *FAP*, *SPP1*, and *MMP11*) as the top markers based on fold-change in patients harboring *SMARCA2* ^Low^ (**Figure 2B**), which indicated particular stromal cells in the TME of these patients. Therefore, we analyzed the distinct composition of cell subpopulations in the TME using multiple algorithms including CIBERSORT, MCP-counter, EPIC, quantiseq, ESTIMATE, and TIMER. Compared to other groups, patients harboring *SMARCA2* ^Low^ & *SMARCA4* ^High^ exhibited higher tumor purity and increased infiltration of cancer-associated fibroblasts (CAFs), lower infiltration of immune cells including CD8^+^ T cells, CD4^+^ T cells (**Figure 2E**). The study by Qi et al. (20) revealed that FAP^+^ fibroblasts and SPP1^+^ macrophages as the desmoplastic niche limit the T cell infiltration in colorectal cancer. We speculated that the similar tumor ecosystem existed in patients harboring *SMARCA2* ^Low^ & *SMARCA4* ^High^.

**Figure 3:**
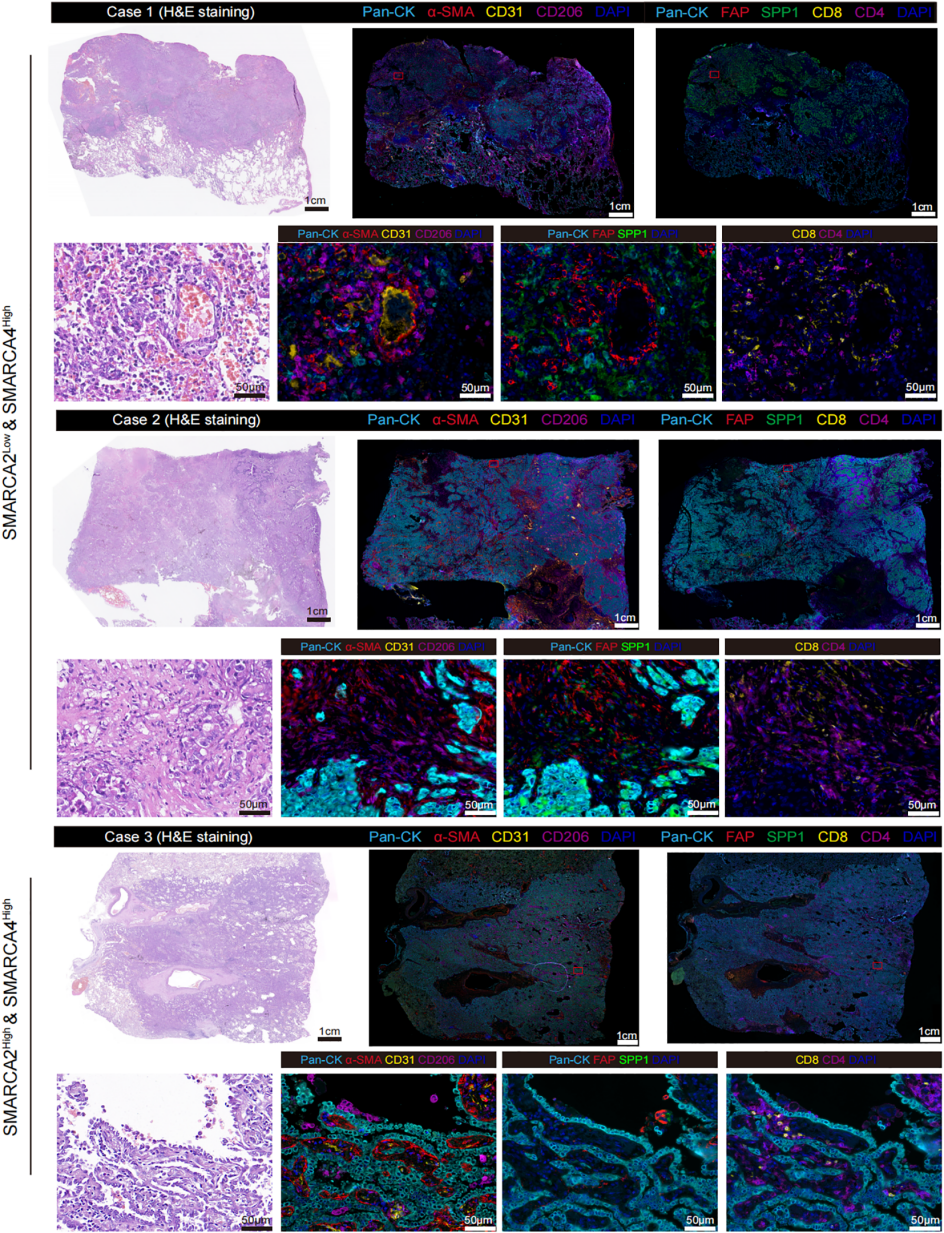
Representative multiplexed IHC staining of SMARCA2 deficiency while preserving SMARCA4 expression samples (n=2) and SMARCA2^High^ & SMARCA2^High^ sample (n=1) stained for panel A: Pan-CK, α-SMA, CD31, and CD206; panel B: Pan-CK, FAP, SPP1, CD8, and CD4.

Next, we analyzed the proteomic LUAD cohort from Chen et al. (n=89) [9]. We found the expression level of SMARCA2 exhibited a significant negative correlation to FAP and SPP1, and a positive correlation to ACTA2, CD3D, CD8A, and CD4 (**Figure 2F**). In contrast, no such relationship was observed in the protein expression of SMARCA4 (**Figure 2F**). To validate our findings from public multi-omics cohorts, we performed multiplexed IHC staining for these two reported samples and one SMARCA2^High^ & SMARCA4^High^ sample as control (**Supplementary Figure S1**) using panel A: Pan-CK (epithelial cells), α-SMA (fibroblasts), CD31 (vessels), and CD206 (macrophages); panel B: Pan-CK, FAP, SPP1, CD8, and CD4 (**Figure 3**). We first observed that the multiple typical stromal components (α-SMA^+^ cells, CD31^+^ cells, and CD206^+^ cells) constituted the neovascularization in tumor tissues, which may establish the prerequisite of intravasation. Moreover, compared to the control sample, we found FAP^+^ fibroblasts and SPP1^+^ macrophages exhibited the co-localization features wrapping around the tumor core and excluded CD8^+^ T cells and CD4^+^ T cells to the stromal region in our reported cases. These results confirmed that the findings from public cohorts and indicated the explicit immunosuppressive TME in these patients.

## Discussion

Herein, we reported the distinct pathological features and poor prognosis in two cases of poorly differentiated LUAD with SMARCA2 deficiency but preserved SMARCA4 expression. This represented a rare subtype of mSWI/SNF complex-deficient tumors. Through multi-omics analysis of public transcriptomic and proteomic datasets, and validation via multiplexed IHC, we discovered that the loss of SMARCA2, coupled with the retention of SMARCA4, was associated with a highly immunosuppressive tumor microenvironment (TME), characterized by the enrichment of FAP^+^ fibroblasts and SPP1^+^ macrophages. The two cells co-localized with CD8^+^ T cells and CD4^+^ T cells, and limited the infiltration of these T cells into the tumor. These findings suggested that the immunosuppressive barrier formed by these stromal cells contributes to poor outcomes in SMARCA2-deficient LUAD.

To further contextualize our findings, we conducted a comprehensive literature review and identified four previously reported cases of SMARCA2-deficient but SMARCA4-preserved tumors with complete clinical descriptions (15, 21). Combined with our two cases, a total of six cases were analyzed and summarized in **Table 1**. Among these, five were located in the thoracic region and one originated from the mesentery. Notably, the cases predominantly occurred in male patients with a history of heavy smoking, and shared common clinical features including poor differentiation, rapid recurrence and poor prognosis. Immunohistochemically, these tumors consistently showed loss of SMARCA2 expression, while SMARCA4 and SMARCB1 remained intact, often accompanied by the loss of other differentiation markers (e.g., TTF-1, p40) and expression of proliferation or EMT-related markers such as Ki-67 and vimentin. Most patients experienced rapid progression and short-term death. This comparative analysis supported the notion that SMARCA2 deficiency, even in the presence of preserved SMARCA4, defines a distinct biological subset of thoracic tumors with dedifferentiated morphology and poor clinical outcomes. Importantly, this group may be under-recognized due to the limited routine assessment of SMARCA2 in thoracic pathology.

**Table 1:** Characteristics of SMARCA2-deficient while SMARCA4-preserved tumors.

ID	Study	Age	Sex	Smoking	Primary Tumor Location	Primary Tumor Size (mm)	Metastatic Site at Diagnosis	IHC staining	Treatment	Follow-up
1	Iwakoshi et al. (15)	42	M	Heavy-smoker	Mediastinum	93	Bone	BRM (-), BRG1 (+), INI1(+), Claudin-4 (-),p40 (-),TTF-1 (-), AE1/AE3 (-), CD34(+).	Chemoradiation therapy	Dead, OS, 8-month
2	Iwakoshi et al. (15)	40	M	Heavy-smoker	Lung>chest wall	85	Adrenal gland	BRM (-), BRG1 (+), INI1(+),Claudin-4 (-), p40 (-),TTF-1 (-), AE1/AE3 (focal +),CD34(-).	Chemotherapy plus ICI, resection for intestinal metastasis	Dead, OS, 10-month
3	Iwakoshi et al. (15)	50	F	Heavy-smoker	Lung	57	Brain	BRM (-), BRG1 (+), INI1(+), p40 (-),TTF-1 (-), AE1/AE3 (+), CD34(-).	Whole-brain radiation followed by chemotherapy	Dead, OS, 2-month
4	Tamaki et al. (21)	70	M	Heavy-smoker	Mesenterium	60	Colon and bladder	BRM (-), BRG1 (+), INI1(+), Vimentin (+),Claudin-4 (-), AE1/AE3 (focal +), CK7 (focal +), Ki-67 (50%).	Surgery	Dead, OS, 20-day
5	Our case	66	M	Heavy-smoker	Lung	32	Mediastinum	BRM (-), BRG1 (+), INI1(+), p40 (-),TTF-1 (-), AE1/AE3 (+), CK7 (+), CgA (-), Ki-67 (70%).	Surgery, Chemotherapy plus ICI	PFS, 11-month
6	Our case	65	M	Heavy-smoker	Lung	30	None	BRM (-), BRG1 (+), INI1(+), p40 (-), TTF-1 (-), AE1/AE3 (+), CK7 (+), CgA (-), Ki-67 (40%).	Surgery, Chemotherapy	Survival, OS, 9-month

As a key epigenetic regulator, the mSWI/SNF complex coordinates gene expression, cell proliferation and differentiation. Loss of expression in multiple subunits of mSWI/SNF complex exhibits common undifferentiated rhabdoid appearances and include malignant rhabdoid tumor as the prototypical example, as well as various other benign and malignant soft tissue tumors in children and adults. In the thoracic tumors, loss of SMARCA4 as the typical form of mSWI/SNF complex-deficient tumors occurs in multiple intrathoracic organs, which is further classified into two subtypes: SMARCA4-dUT and SMARCA4-dNSCLC. The relationship between the two subtypes remains elusive. These two groups of SMARCA4-deficient tumors were separable morphologically and immunohistochemically, but both SMARCA-4 dUT and SMARCA4-dNSCLC are associated with heavy smoking, male preponderance, and poor prognosis. Notably, the loss of both SMARCA-4 and SMARCA2 was relatively specific to SMARCA4-dUT compared with SMARCA4-dNSCLC. Rekhtman et al. (13) proposed a model that the dedifferentiation process of NSCLC was activated by loss of SMARCA2 in the context of SMARCA4-deficient expression. Besides, a previous study showed that SMARCA2 deficiency with preserved SMARCA4 promotes lung tumors initiation and development using mouse models (8). Similarly, in alveolar rhabdomyosarcoma (ARMS), SMARCA4 is preferentially expressed over its mutually exclusive homolog SMARCA2 and is rarely subject to mutation or deletion. Besides, SMARCA4 is required for the expression of the oncogenic fusion protein PAX3:FOXO1, thereby promoting chemotherapy resistance and long-term tumor cell survival, despite being dispensable for short-term viability (22). Collectively, these findings underscored the context-dependent and non-redundant roles of SMARCA4 and SMARCA2 in tumor development, progression, and therapeutic response across a spectrum of SWI/SNF-deficient malignancies, highlighting their potential as both diagnostic markers and therapeutic targets.

In our report, all these two cases were heavy smoking male patients and exhibited the poorly dedifferentiation features with SMARCA2 deficiency with preserved SMARCA4. In these cases, Case 1 showed rapid intrathoracic recurrence after undergoing both surgical treatment and postoperative adjuvant chemotherapy, which indicated that the specific carcinogenic pathway activation in these patients. Through the multi-omics analysis, SMARCA2^Low^ patients (including *SMARCA2* ^Low^& *SMARCA4* ^High^ and *SMARCA2* ^Low^& *SMARCA4* ^Low^) harboring shorted OS and RFS exhibited the enrichment in the tumorigenicity including cell nuclear division, G2/M checkpoint, and EMT, which is consistent with previous understanding of the function of mSWI/SNF complex. Surprisingly, FAP and SPP1 as the noticeable markers in DEGs showed a negative correlation with the protein level of SMARCA2. Smoking history exhibited a close relationship with FAP expression in fibroblasts and SPP1 expression in lung antigen-presenting cells (APCs) (23, 24). Mechanically, benzo[a]pyrene as a carcinogen in cigarette smoke induces higher expression levels of fibrosis (FAP, α‐SMA, and β‐catenin) in fibroblasts through hepatocellular carcinoma exosome‐circular RNA (25). In addition, cigarette smoke could directly induce activation of SPP1 in lung APCs and further exacerbate emphysema (24). Besides, previous study reported that the formation of FAP^+^ fibroblasts and SPP1^+^ macrophages also rely on the activation of TGF-β, which further induced the fibrotic structure that restricted T cells infiltration and predicted the immunotherapy resistance (20). In our reported cases, we demonstrated that FAP^+^ fibroblasts and SPP1^+^ macrophages as the contributor to low immune cell infiltration in SMARCA2 deficiency with preserved SMARCA4 poorly LUAD. Our cases, together with similar findings in the TME of colorectal cancer, may lead to new strategies for normalizing tumor immune microenvironment.

Therapeutic strategies specifically tailored for SMARCA2-deficient while SMARCA4-retained tumors remain limited, largely due to the rarity and heterogeneity of these tumors. However, several potential interventions have been proposed based on emerging molecular insights. First, the preserved expression of SMARCA4 suggests partial retention of SWI/SNF complex function, distinguishing these tumors from SMARCA4-deficient thoracic cancers that are typically associated with extremely poor prognosis and resistance to standard therapies. Second, the frequent co-occurrence of mutations in tumor suppressor genes such as STK11, ARID1A, and CDKN2A, as observed in our case, may inform potential vulnerabilities to targeted therapies, including mTOR/AMPK inhibitors, CDK4/6 inhibitors, or agents modulating epigenetic regulators (26, 27). Third, elevated PD-L1 expression and intermediate-to-high TMB in these patients support the potential efficacy of immune checkpoint inhibitors (ICIs), although clinical outcomes appear variable and may depend on co-existing immunosuppressive mechanisms. In this context, combination strategies incorporating ICIs with SPP1 or FAP inhibitors may hold promising therapeutic potential by targeting both immune evasion and stromal-mediated immunosuppression. Lastly, preclinical studies have shown that synthetic lethality targeting residual SWI/SNF function (e.g., via EZH2, BRD9, or ATR inhibition) could be leveraged in SMARCA2-deficient tumors (28). Moreover, proteolysis-targeting chimeras (PROTACs) directed against SMARCA4, and HDAC inhibitors (e.g., trichostatin A) capable of reactivating epigenetically silenced SMARCA2 (29), are being explored as potential treatments in tumors with functional SMARCA2 loss rather than genetic deletion. These findings underscore the importance of comprehensive molecular profiling to guide personalized treatment approaches in this distinct molecular subset of LUAD.

While our study provides novel insights into the roles of SMARCA2 and SMARCA4 in LUAD pathogenesis, there are several limitations. First, the sample size was limited to two cases, which may affect the generalizability of the findings. Larger studies with additional cases of SMARCA2-deficient LUAD are needed to confirm the clinical and biological relevance of our observations. Second, although we utilized multi-omics datasets for validation, direct functional studies were not conducted to establish the causal relationship between SMARCA2 deficiency, TME remodeling, and immune exclusion. Future studies should aim to characterize the broader prevalence and clinical significance of SMARCA2 deficiency in NSCLC. Given the association between SMARCA2 loss and an immunosuppressive TME, therapeutic strategies targeting FAP^+^ fibroblasts or SPP1^+^ macrophages may hold promise for overcoming immune evasion in this tumor subtype. Additionally, investigating whether SMARCA2-deficient tumors exhibit differential responses to immune checkpoint inhibitors or other immunotherapies could provide further insights into personalized treatment approaches for these patients. Lastly, future research should explore the interplay between SMARCA2 and SMARCA4 in greater detail, as the preservation of SMARCA4 may modulate the biological behavior of SMARCA2-deficient tumors in ways that are distinct from dual-deficient tumors.

## Methods

### Public datasets collection and bioinformatic analysis

The bulk RNA-seq dataset of LUAD with corresponding histopathological information was derived from the Gene Expression Omnibus (GEO) with accession numbers GSE31210 (n=226) (19). This data normalization process and DEGs analysis were conducted using the R package “limma” (v3.54.1). DEGs were considered for further analysis with adjusted P-value < 0.05 and absolute log2(FoldChange) > 0.585. The over-representation analysis of Gene Ontology (GO) was performed using the R package “clusterProfiler” (v4.6.0). We performed Gene Set Enrichment Analysis (GSEA) (30) to examine the hallmark gene sets (MSigDB, https://www.gsea-msigdb.org/gsea/msigdb) that are significantly enriched pathway. The proteomics dataset of LUAD derived from the study by Chen et al. (n=89) (31). We employed a multi-faceted approach (including CIBERSORT (32), MCP-counter (33), EPIC (34), ESTIMATE (35), quantiseq (36), and TIMER (37)) to evaluate the diversity in the TME. The above algorithms are included in the R package “IOBR” (v0.99.9) (38).

### Next-generation sequencing

Tumor tissues were snap-frozen in liquid nitrogen immediately after collection. Each sample measured less than 1.5 cm × 1.5 cm, with a thickness of 2-5 mm. Genomic DNA was extracted from fresh-frozen specimens using the QIAamp DNA Mini Kit (Qiagen, 51306, Valencia, CA, USA) following the manufacturer’s instructions. DNA libraries were prepared using the KAPA Hyper Prep Kit (KAPA, KK8504) and enriched with the Agilent SureSelect XT Human All Exon V5 Kit (Agilent Technologies, Santa Clara, CA, USA). Targeted sequencing of exons from major cancer-related genes was conducted using Genetron’s 509-gene cancer panel on the Illumina NovaSeq 6000 platform, with matched white blood cell (WBC) DNA used as a germline control.

### H&E and multiplexed IHC staining

Representative 4-μm-thick slides of FFPE tissue were stained for H&E and checked by 2 certified pathologists to determine histological patterns. Multiplexed IHC staining was performed using Novo-light 5-color kit (D110051-50T, WiSee Bio) according to the manufacturer’s protocol. Briefly, deparaffinized slides were incubated with various primary antibodies, followed by treated with horseradish peroxidase-conjugated secondary antibody incubation and tyramide signal amplification working solution. Between all steps, the slides were washed with buffer. Finally, nuclei were subsequently visualized with DAPI, and the slides were coverslipped using an anti-fade mounting medium. Multiplexed IHC was performed with the same protocols but different primary antibodies for two panels: Panel A: anti-Pan-CK (BX50143, Biolyx, 1:1000), anti-α-SMA (BM0002, Boster, 1:1000), anti-CD31 (BX50032-C3, Biolyx, 1:300), and anti-CD206 (91992, CST, 1:200); Panel B: anti-Pan-CK (BX50143, Biolyx, 1:1000), anti-FAP (BM5121, Boster, 1:250), anti-SPP1 (ab214050, Abcam, 1:2000), anti-CD8 (BX50036, Biolyx, 1:300), and anti-CD4 (BX50023, Abcam, 1:300). For each patient specimen, whole-slides were scanned using Pannoramic MIDI platform.

### Statistical analyses

All statistical analyses and graphical presentations were performed using open-sourced R (v4.2.2). Survival analysis was performed using the R packages “survminer” (v3.1-8) and “survival” (v3.1-8). Patients within all datasets were divided into two groups based on the median of gene expression, and P-value was calculated using a log-rank test. Correlation analysis was created with Pearson’s correlation. The statistical tests used in the figures are specified in the figure legends, and statistical significance was set at a P-value < 0.05.

## Ethics approval

This study was approved by the institutional review board (PJ-KS-KY-2023-362). All patients had signed informed consent for the inclusion of their clinical information and specimens in research projects, following the recommendation of the ethical committee of the First Affiliated Hospital of Dalian Medical University. Written informed consent was obtained from the participant/patient(s) for the publication of this case report.

## Data Availability

The original contributions presented in the study are included in the article/**Supplementary Material**. Further inquiries can be directed to the corresponding author.
