# Supplementary figures and images for: Case Report: FAP+ fibroblasts and SPP1+ macrophages in SMARCA2-deficient while SMARCA4-preserved poorly differentiated lung adenocarcinoma: two case reports and multi-omics analysis
Source: Front Immunol. 2025 May 16;16:1568556. doi: 10.3389/fimmu.2025.1568556 (PMC12122539; doi:10.3389/fimmu.2025.1568556)

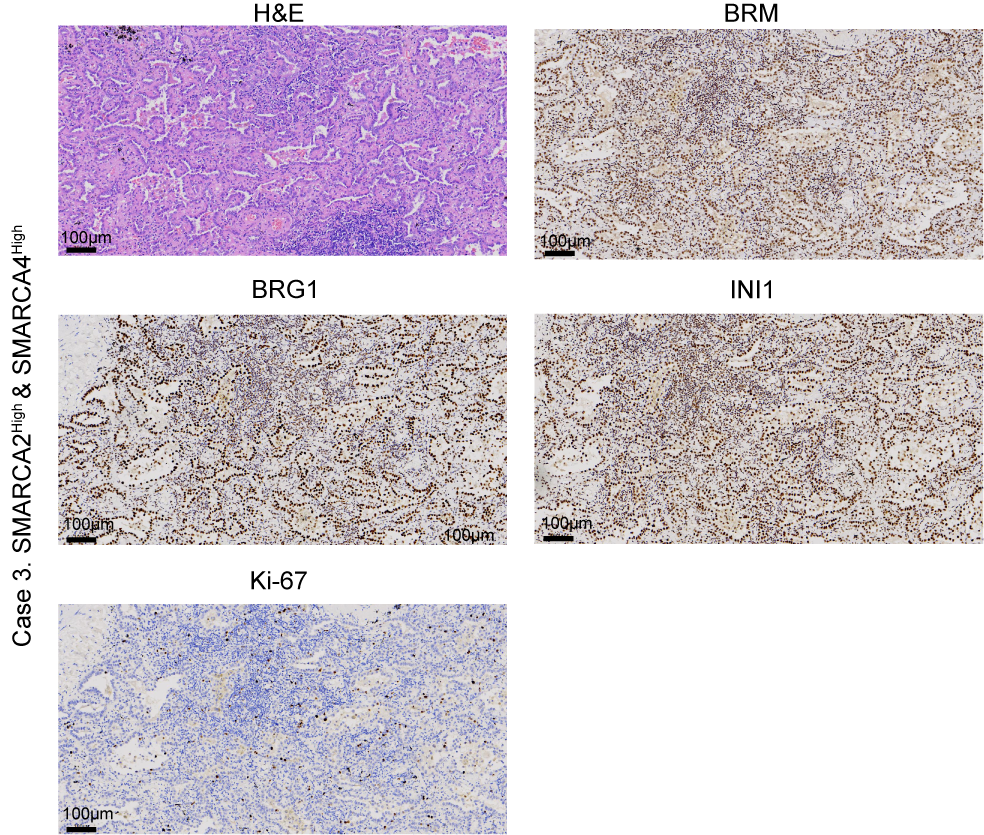

Supplement: Supplementary file 1 [file Image1.tif]
